# Obtaining long-term stage-specific relative survival estimates in the presence of incomplete historical stage information

**DOI:** 10.1038/s41416-022-01866-8

**Published:** 2022-06-17

**Authors:** Rachael Stannard, Paul C. Lambert, Therese M.-L. Andersson, Mark J. Rutherford

**Affiliations:** 1 Biostatistics Research Group, Department of Health Sciences, University of Leicester, Leicester, UK; 2 Department of Medical Epidemiology and Biostatistics, Karolinska Institutet, Stockholm, Sweden

**Keywords:** Epidemiology, Cancer epidemiology

## Abstract

**Background:**

Completeness of recording for cancer stage at diagnosis is often historically poor in cancer registries, making it challenging to provide long-term stage-specific survival estimates. Stage-specific survival differences are driven by differences in short-term prognosis, meaning estimated survival metrics using period analysis are unlikely to be sensitive to imputed historical stage data.

**Methods:**

We used data from the Surveillance, Epidemiology, and End Results (SEER) Program for lung, colon and breast cancer. To represent missing data patterns in less complete registry data, we artificially inflated the proportion of missing stage information conditional on stage at diagnosis and calendar year of diagnosis. Period analysis was applied and missing stage at diagnosis information was imputed under four different conditions to emulate extreme imputed stage distributions.

**Results:**

We fit a flexible parametric model for each cancer stage on the excess hazard scale and the differences in stage-specific marginal relative survival were assessed. Estimates were also obtained from non-parametric approaches for validation. There was little difference between the 10-year stage-specific marginal relative survival estimates, regardless of the assumed historical stage distribution.

**Conclusions:**

When conducting a period analysis, multiple imputation can be used to obtain stage-specific long-term estimates of relative survival, even when the historical stage information is largely incomplete.

## Background

Missing cancer stage information is a common issue for cancer registration data, and the proportion of this missing information often varies dramatically by calendar year. Generally, the completeness of stage at diagnosis increases over time since many cancer registries have made substantial improvements in recording in recent years. For some cancer registries, such as in England, it has only been possible to provide short-term survival estimates that are stratified by stage at diagnosis [1–4]. However, estimating longer-term survival estimates stratified by stage, and particularly estimating stage-specific life expectancy estimates would be of interest.

There are examples of countries with low national completeness of stage where there exist regions that have historical better completeness. Often, this sub-national data is used to provide longer-term stage-specific survival estimates [5]. However, longer-term stage-specific survival measures at a whole population level are preferential. These long-term estimates of survival or derived metrics such as stage-specific life expectancy have been possible in other locations thanks to historical better completeness [6, 7]. It would be useful to develop a consistent approach to estimation in the case of historical high incompleteness of stage information.

Multiple imputation offers one approach to obtain long-term stage-specific survival measures in the presence of poor historical stage at diagnosis information. There have been a number of methods proposed for fitting appropriate imputation models in a cancer registry context [8–12], some of which have focussed on the different missing data mechanisms over calendar time [13].

Another feature to consider when estimating long-term survival measures, is that it is often necessary to account for improvements in survival over calendar time. This is usually achieved either by modelling calendar period, or more commonly through the use of a period analysis [14]. Implementing a period analysis restricts which patients contribute to the survival metrics over time. Only patients diagnosed recently contribute to the short-term metrics, resulting in more up-to-date survival estimates.

Baseline cancer-specific excess mortality rates are greatest shortly after diagnosis and decrease with time [15, 16]. Much of the difference in survival due to the effect of stage at diagnosis is captured by the differences in short-term prognosis. The effect of stage at diagnosis on excess mortality rate is time-dependent and diminishes with time [3, 15]. For individuals who survive longer, the long-term impact of stage at diagnosis on survival is less pronounced, conditional on them surviving for a given time period post-diagnosis. Given the issue of historical poor recording of stage in cancer registries, but the desire to provide long-term survival metrics, an assessment of the impact of the various choices that can be made would be of interest. We take advantage of these features in the approach we outline for multiple imputation to obtain reliable long-term stage-specific survival estimates.

In this paper, we evaluate various approaches of estimating stage-specific relative survival when the historical stage completeness is poor. We use SEER data (which has historically good recording of SEER summary stage at diagnosis) under conditions where we artificially inflate the proportion of missing stage at diagnosis information to assess different assumptions. With the highly complete registry data, we further show the stage profile of individuals who contribute to the long-term survival estimates, which abates some of the concerns over the appropriate imputation approach of the historical data. In doing so, we show that for registries with historical incompleteness, but recent good recording of stage at diagnosis, under which conditions reliable stage-specific estimates of relative survival can be achieved.

## Methods

### Data

Data were obtained from the US Surveillance, Epidemiology and End Results (SEER) Programme [17] to investigate the possible impact of the chosen imputation model for cancer stage at diagnosis on marginal relative survival estimates. Data were extracted for patients diagnosed between 2005 and 2017. A period analysis was applied so that only recently diagnosed patients contribute to the short-term estimates, and patients who were diagnosed several years ago only contribute to the long-term estimates [18]. A standard cohort approach includes the short-term survival experiences of patients who were not recently diagnosed in the estimation of short-term survival. This method produces less up-to-date survival estimates, especially for cancer sites with advances in survival in recent years. The period window spanned from January 1, 2015 to December 31, 2017, with follow-up restricted to 10 years.

We included three cancer sites (lung, colon and breast) which cover poor, moderate and good prognosis cancer sites respectively. In this paper, we primarily report the results for colon cancer patients, with results for lung cancer and breast cancer patients reported in the appendices. Patients diagnosed with colon cancer were identified using the primary tumour site International Classification of Diseases for Oncology, third edition (ICD-O-3) codes C18.0–C18.9. Similarly, lung cancer cases were identified with ICD-O-3 codes C34.0-C34.9 and breast cancer cases with C50.0-C50.9. Cancer stage at diagnosis is coded as localised, regional marginal or missing as defined by the SEER summary stage classification [19]. Grade is coded as I, II, III, IV or missing. Sex is coded 1 for females and 0 for males. The integer value of age is available for all subjects aged 99 years or younger.

As stated, these cancer sites typically yield worse and better survival respectively compared to the experiences of colon cancer patients, and hence will provide a broader view of the possible impact of the assumptions. The methods described below were applied to each cancer site in turn.

### Missing stage at diagnosis information

**Fig. 1:**
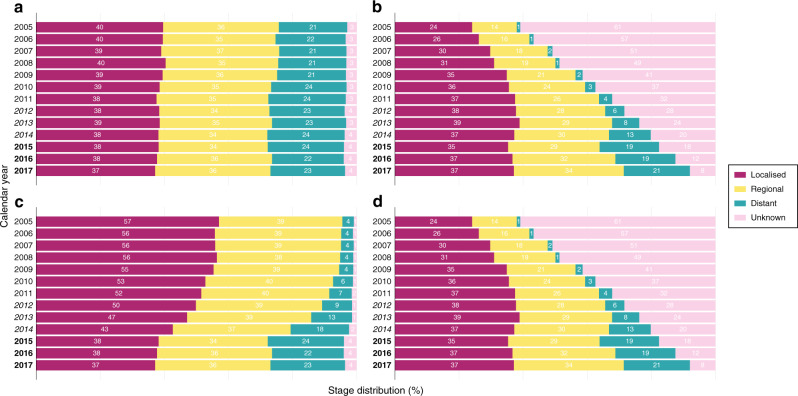
Distribution of stage at diagnosis over calendar time. Stage distribution among colon cancer patients observed over time for **a** the whole cohort diagnosed from 2005 to 2017, **b** the whole cohort with increased missing stage information, **c** the period cohort (period window 2015–2017) and **d** altered period cohort with increased missing stage information.

The US SEER data is highly complete for SEER summary stage: 96.72% for colon cancer, 97.7% for lung cancer and 99.00% for breast cancer. For patients experiencing follow-up within the period window from January 1, 2015 to December 31, 2017, this increased to 97.84, 98.32 and 99.34% for each cancer site, respectively. To investigate the effect of extreme imputation conditions, we artificially increased the proportion of missing stage information. This change also allows the data set to better represent the missing data patterns present in other populations. However, the proportion of missing information does not indicate whether multiple imputation should be used [20]. The designed missing data mechanism is conditional on both calendar year and stage at diagnosis and is illustrated in Fig. 1b. We artificially increased the proportion of missing stage information by changing 35% of patients diagnosed with regional cancer in 2011 to missing stage. This proportion increased in 5% increments with every calendar until it reached 65% in 2005. Patients with distant cancer were 7 percentage points more likely to have their stage information artificially removed than patients diagnosed with regional cancer in the same calendar year (42% of patients with localised stage changed to missing stage in 2011 and 72% of patients with distant stage changed to missing stage in 2005). Patients with localised cancer were 7 percentage points less likely to have their stage information artificially removed than patients diagnosed with regional cancer in the same calendar year (28% of patients with localised stage changed to missing stage in 2011 and 58% of patients with distant stage changed to missing stage in 2005). We wish to show that the method is still effective when the recent data is not as highly complete, and hence the proportion of missing stage data in the years 2012–2017 was also increased by 20 percentage points. The missing data mechanism in these years is not conditional on stage or calendar year. This is a plausible range of missingness for many registries during this calendar year range particularly. The SEER data are very complete, but data from other jurisdictions have much lower completeness. For instance, for non-small cell lung cancer in 2010–2014 the UK registries have a 33% missingness proportion for TNM stage, with New Zealand showing a similar proportion of missingness for SEER summary stage [21–23]. Particularly for the UK, the completeness of stage increases dramatically in 2012.

### Multiple imputation

For all individuals included in the period analysis with unknown cancer stage at diagnosis or those whose stage information was artificially removed, cancer stage was imputed with 30 iterations under a multinomial logistic model [24]. This imputation model consisted of sex, age, subsite, cancer grade at diagnosis, calendar year of diagnosis, the event indicator and the Nelson–Aalen estimate of cumulative hazard (*H*), *H*_1_, interactions between sex and *H*, age and *H*, sex and *H*_1_ and age and *H*_1_ as recommended by the time-varying effects approximate approach of Keogh and Morris [9, 25, 26]. As described by Keogh and Morris, *H*_1_ is similar to the Nelson–Aalen cumulative hazard estimator and is defined as ${\it{H}}_1\left( {\it{t}} \right) = \mathop {\sum}\nolimits_{t \le T} {t\frac{{d(t)}}{{n(t)}}}$, where *d*(*t*) and *n*(*t*) are the number of deaths and number at risk at time *t*. The event indicator is 1 for non-events and 2 in the case of death due to any cause. Cancer grade at diagnosis was not known for all patients, but for patients with an observed grade and stage, the grade appeared highly correlated with cancer stage at diagnosis. Hence, it was included in the imputation model with an unknown category. Subsite information was modelled categorically using the ICD-O-3 codes and calendar year was modelled categorically as integer values. Cancer grade at diagnosis, subsite and calendar year of diagnosis were included as auxiliary variables.

### Flexible parametric survival model

For each of the 30 imputed data sets, a flexible parametric survival model was fitted on the cumulative excess hazard scale, with a separate model for each cancer stage [27]. This enables different covariate effects to be estimated for each cancer stage. The baseline cumulative excess hazard was modelled using restricted cubic splines with 5 degrees of freedom. Restricted cubic splines allow complex baseline cumulative excess hazard functions to be captured without having to make strong distributional assumptions. The selected flexible parametric relative survival model included age and sex. Age was modelled using restricted cubic splines with 4 degrees of freedom with the effect due to age constrained to be constant in the upper and lower 2 percentiles of the data [28]. Constraining the data in this way produces more stable estimates in the tails where the data is often sparse. Time-dependent effects were enabled for each covariate using splines with 2 degrees of freedom. New restricted cubic splines to model the time-dependent effect of age were calculated here with just 2 degrees of freedom, rather than 4 as before. Expected population mortality rates were also obtained from the SEER Programme and are stratified by calendar year, sex and age. Once a subject reaches 99 years of age, the expected mortality rate at this point is carried over for as many years as required.

The stage-specific marginal relative survival was estimated for 10 years of follow-up, and age-standardised to the marginal age distribution of patients diagnosed in 2017. The marginal excess hazard was estimated similarly [29]. The estimates of marginal relative survival and marginal excess hazard from the 30 data sets were then combined using Rubin’s Rules [30].

### Pre-window and period window

**Fig. 2:**
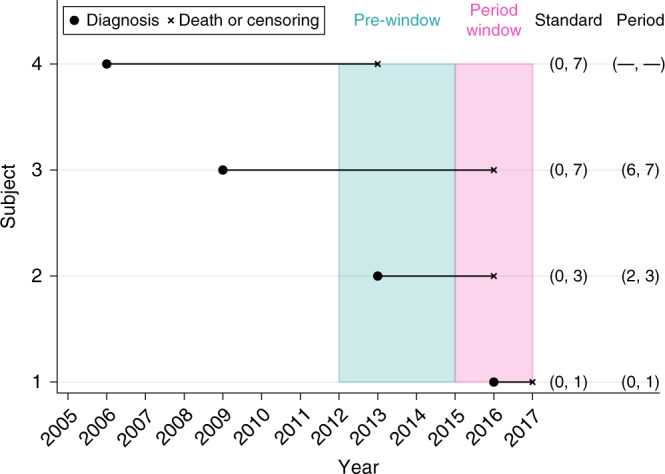
Illustration of the period window (2015–2017) and the pre-window (2012–2015). The bracketed intervals compare the years of follow-up from each subject that contribute to the analysis under the standard approach and the period analysis approach.

To improve the stability of the estimated survival metrics, we introduce a pre-window period. This restricts which patients are affected by strong imputation assumptions to only those diagnosed more than a few years before the period window. The pre-window reflects a portion of time immediately before the period window in which stage information is more complete. Figure 2 provides a graphical representation of the period window and pre-window. In this particular setting, the pre-window (2012–2015) provides three years of sufficiently complete data prior to the period window. The aim is to improve the stability of the estimated survival metrics following multiple imputation. Subjects 1–3 would be included in the period analysis since they experience follow-up during the period window 2015–2017; however, subject 4 would not be included as they were censored in 2013 and therefore do not experience any follow-up during the period window.

### Multiple imputation conditions

For each cancer site, a series of conditions were applied to the imputation model in order to reflect the most extreme stage distributions following multiple imputation and compare the associated survival estimates to standard case. It is crucial to note that these conditions are not the recommended approach for multiple imputation but are merely used to illustrate the stability of the relative survival estimates regardless of the chosen imputation approach. They emulate the most extreme assumptions that could be made regarding the historical stage distribution.

The conditions are as follows:

**Condition 1:** First, the stage of all individuals with follow-up during the period window and missing stage at diagnosis was imputed using the standard multiple imputation approach. This applies to all individuals with unknown stage at diagnosis in the original data set and those whose stage information was artificially removed.

**Condition 2:** Under the second condition, stage at diagnosis was only imputed for individuals with unknown stage at diagnosis diagnosed on or after the start of the pre-window in 2012. Complete case analysis was applied to individuals diagnosed before this pre-window, hence only individuals with non-missing stage at diagnosis were included in the flexible parametric model.

**Condition 3:** Similar to condition 2, stage was only imputed for individuals diagnosed on or after the pre-window. However, individuals diagnosed before the pre-window with unknown stage at diagnosis were assumed to have localised cancer.

**Condition 4:** As with conditions 2 and 3, stage was only imputed for individuals diagnosed on or after the pre-window. However, individuals diagnosed before the pre-window with unknown stage at diagnosis were assumed to have distant cancer.

These conditions were applied again, but this time without the pre-window. Hence, the conditions were not only applied to patients diagnosed before 2012 but also to those diagnosed up until the start of the period window in 2015. In addition to increasing the number of patients subject to the conditions, removing the pre-window is also likely to increase the impact of the conditions due to the shift in stage distribution, and the assumptions applying also to follow-up time shortly after diagnosis.

Figure 2 illustrates the pre-window period and the period window. We will discuss the implications of the pre-window and imputation conditions 1–4 on each of the subjects 1–4 in turn, under the assumption that the stage information for subjects 1–4 is missing. Under each of the conditions 1–4, and regardless of the pre-window, subject 1 would be imputed as usual as they are diagnosed after the start of the period window in 2015. In the presence of the pre-window, subject 2 is imputed as usual under each condition 1–4 since they are diagnosed after the start of the pre-window in 2012. However, without the pre-window, when considering the complete case analysis approach in condition 2, they would be excluded from the analysis since they are diagnosed before the start of the period window. For similar reasons, under conditions 3 and 4 we would assume they were diagnosed with localised and distant stage respectively. Subject 3 would always be excluded under condition 2 regardless of whether the pre-window is implemented since they are diagnosed before the start of the pre-window. Similarly, we would always assume they were diagnosed with localised and distant stage for conditions 3 and 4, respectively. Lastly, subject 4 would never be included in the analysis since they do not experience any follow-up during the period window at all.

To ensure any differences in stage-specific marginal relative survival across the multiple imputation models are not due to the assumptions of the survival model, the results were compared to non-parametric estimates obtained using the Pohar–Perme method [31, 32]. Using the five International Cancer Survival Standard (ICSS) age groups (<45, 45–54, 55–64, 65–74, 75+) [33], the estimates were internally age-standardised to the age distribution of SEER patients diagnosed in 2017.

All analyses were conducted using Stata 17 [34], and the code is available at 10.25392/leicester.data.19383518.

## Results

Table 1 shows the number (%) of colon patients in each ICSS age group and each cancer stage at diagnosis by sex. Overall, the proportion of males and females are similar; however, there were noticeably more females than males aged over 75 years. The stage distribution among females and males was very similar. The mean (SD) age at diagnosis is also given by sex and stage at diagnosis, with those with missing stage information being older on average (Table 1).

**Table 1:** Baseline characteristics for the whole cohort of colon cancer patients diagnosed from 2005 to 2017, prior to applying a period analysis.

	Sex					
	Female		Male		Total	
*Total*	54,299	(51.13%)	51,892	(48.87%)	106,191	(100.00%)
*Age group at diagnosis*						
<45	2998	(5.52%)	2838	(5.47%)	5836	(5.50%)
45–54	6263	(11.53%)	7108	(13.70%)	13,371	(12.59%)
55–64	9358	(17.23%)	11,653	(22.46%)	21,011	(19.79%)
65–74	12,297	(22.65%)	13,463	(25.94%)	25,760	(24.26%)
>75	23,383	(43.06%)	16,830	(32.43%)	40,213	(37.87%)
*Stage at diagnosis*^a^	(*n* = 52,282)		(*n* = 50,224)		(*n* = 102,506)	
Localised	20,631	(39.46%)	20,399	(40.62%)	41,030	(40.03%)
Regional	19,500	(37.30%)	17,897	(35.63%)	37,397	(36.48%)
Distant	12,151	(23.24%)	11,928	(23.75%)	24,079	(23.49%)
Missing	2017	(3.71%)	1668	(3.21%)	3685	(3.47%)
*Mean (SD) age at diagnosis*						
Stage at diagnosis						
Localised	69.45	(14.72)	67.16	(13.61)	68.32	(14.23)
Regional	70.01	(14.65)	66.99	(13.79)	68.57	(14.33)
Distant	68.02	(15.06)	65.60	(14.01)	66.82	(14.60)
Missing	79.81	(13.63)	74.10	(14.53)	77.23	(14.33)

^a^Stage proportions calculated from the observed distribution.

**Fig. 3:**
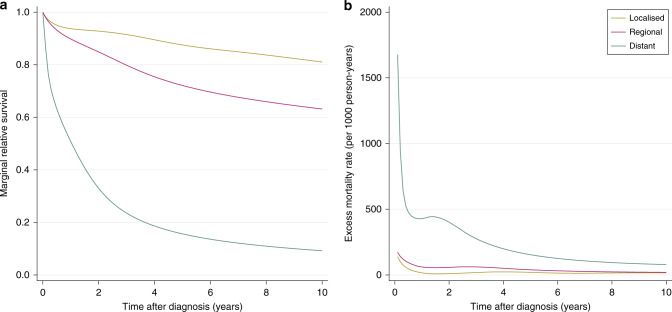
Estimated survival metrics for the full cohort of colon cancer patients diagnosed from 2005 to 2017. Marginal relative survival is given in panel (**a**). Excess hazard is given in panel (**b**).

The stage-specific marginal relative survival and excess hazard of colon cancer patients is illustrated in Fig. 3. These estimates were obtained from the full data set without simulating additional missing stage information, and the stage of the few subjects with unknown stage was imputed as described in condition 1. Figure 3a shows the vast difference in prognosis between patients with localised or regional cancer and those with distant cancer, whose prognoses are very poor. The greatest decrease in relative survival is seen in the years immediately after diagnosis. The same trends are observed in Fig. 3b, which further highlights the worse survival experienced by patients diagnosed with distant cancer at diagnosis, and that the stark differences in prognosis are greatest in the months directly after diagnosis.

The observed stage distribution by calendar year for the whole cohort of colon patients is presented in Fig. 1. The stage distribution and proportion of missing data in the original data set is stable over time, as shown in Fig. 1a. Figure 1c displays the stage distribution of patients included in the period analysis. Compared to Fig. 1a, the stage distribution of patients diagnosed before the period window has shifted. There is now a greater proportion of patients with localised and regional cancer and fewer patients with unknown cancer stage. Figure 1b illustrates the effect of simulating missing stage information. The simulation increases the proportion of missing stage information within the period window at random conditional on stage and calendar year of diagnosis for patients diagnosed before the period window. Figure 1d displays the final cohort after applying a period analysis and increasing the proportion of missing stage information.

**Fig. 4:**
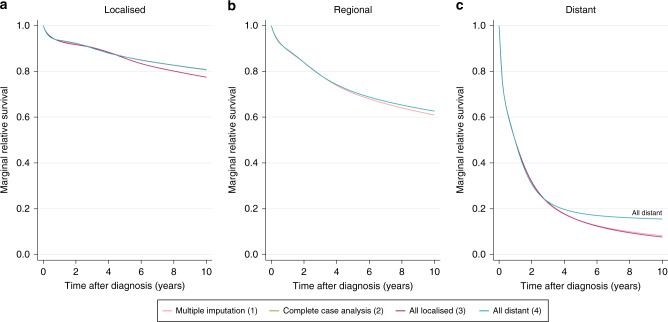
Estimated stage-specific marginal relative survival with the pre-window (2012–2015) for colon cancer patients who experienced follow-up during the period window (2015–2017). (1)–(4) refer to imputation conditions (1)–(4). Panels (**a**–**c**) provide estimates for patients diagnosed with localised, regional and distant stage colon cancer respectively.

The estimated stage-specific marginal relative survival following multiple imputation is shown in Fig. 4. The estimates shown in this figure were obtained from the data where the proportion of missing stage information is artificially increased. They are marginally lower than the estimates obtained from the full data set, which represent the underlying case (Table A1). The pre-window approach has been implemented, hence all subjects diagnosed on or after 2012 have been imputed as usual under each of the four conditions. Despite the extreme nature of the imposed imputation conditions, each of the four approaches yield similar estimates The most prominent deviation from the underlying estimates exists for the 10-year marginal relative survival estimate for distant cancer patients, under condition 4 where patients with unknown stage at diagnosis were recoded to have distant cancer. This result is labelled in Fig. 4c. Subjects whose underlying, but unknown, stage at diagnosis is regional or localised contribute to the distant-stage marginal relative survival estimate. The survival of these patients is expected to be better than the survival of subjects whose underlying stage at diagnosis is distant, hence the distant-stage marginal relative survival estimate is inflated. Each of the remaining imputation conditions achieve a high level of agreement for each stage at diagnosis. The “Complete Case Analysis (2)” line has been completely overlapped with another line in each subplot of Fig. 4. This is with “All Distant (4)” in Fig. 4a, “All Localised (3)” and “All Distant (4)” in Fig. 4b and “All Localised (3)” in Fig. 4c. The estimated stage-specific marginal relative survival is also given at 1, 5 and 10 years for each imputation condition in Table A1 in the appendices. These results are presented again in Figure A1 in the appendices, alongside the estimates obtained by the Pohar–Perme non-parametric approach. These estimates are consistent with those obtained using flexible parametric models.

**Fig. 5:**
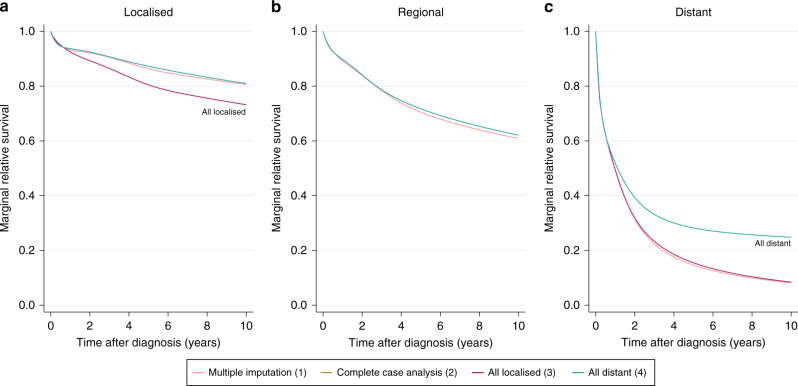
Estimated stage-specific marginal relative survival without the pre-window for colon cancer patients who experienced follow-up during the period window (2015–2017). (1)–(4) refer to imputation conditions (1)–(4). Panels (**a**–**c**) provide estimates for patients diagnosed with localised, regional and distant stage colon cancer respectively.

Figure 5 displays the stage-specific marginal relative survival, but now without the pre-window. Without the pre-window, the distant-stage marginal relative survival estimate obtained by imputation condition 4 cancer deviates further from the other imputation approaches, as highlighted on Fig. 5c. Figure 5a illustrates a similar deviation; the estimate for localised patients is decreased when assuming all subjects with unknown stage at diagnosis have localised cancer, as described by imputation condition 3. Without the pre-window, we allow very extreme assumptions to be made regarding the stage distribution of patients whose underlying stage at diagnosis is unknown. Removing the pre-window leads to a further 85,566 patients with unknown stage at diagnosis, and therefore increasing the number of patients for whom we assume localised or distant stage at diagnosis by 39 percentage points. A number of these patients have been very recently diagnosed and hence it is possible that we are allocating a large number of patients to the localised group who only survive a short while after diagnosis. This lead to the localised-stage marginal relative survival to be underestimated.

This analysis was repeated for the lung cancer data set as shown in Table B1 and Figs. B1–B3 in the appendices. We see a high level of agreement between each of the imputation conditions.

The same methods were also applied to the breast cancer data set. The baseline characteristics and results can be seen in Table C1 and Figs. C1–C3 in the appendices. Generally, there is a high level of agreement between each of the conditions as with the lung cancer data set, with the exception of the marginal relative survival estimate for distant patients when we assume all patients with missing cancer stage at diagnosis have distant cancer.

## Discussion

Using the SEER colon cancer data as an illustrative example, we have shown that stage-specific estimates of marginal relative survival are largely robust to variations in the imputation of stage at diagnosis. Although there is some discrepancy between the distant-stage marginal relative survival estimates, the imputation conditions emulate the most extreme stage distributions possible. Hence, we expect the marginal relative survival estimates obtained by any reasonable imputation model are not likely to deviate beyond what we have observed. Imputation conditions 3 and 4 assume that patients diagnosed before the pre-window whose stage information is missing were diagnosed with diagnosed with localised and marginal cancer respectively. We do not recommend these approaches in practice due to the strong and unrealistic assumptions they make regarding the stage distribution, however the stability of the marginal relative estimates under these assumptions provides a good level of confidence when imputing historical stage at diagnosis information.

The chosen imputation model does not radically alter the stage-specific marginal relative survival estimates largely since the differences in survival due to cancer stage at diagnosis are most pronounced in the years that immediately follow a diagnosis, and the mortality rate tends to be greatest in the short term. Additionally, the application of the period analysis restricts the years of follow-up that contribute to the estimates from each individual. Hence, only recently diagnosed individuals contribute to the short-term estimates. Combining these two notions results in the short-term data having the greatest impact on survival estimates, even for long-term estimates. Since the completeness of stage at diagnosis information is good in the short term, the marginal relative survival estimates remain stable when the stage distribution of the long-term data is altered.

We have demonstrated the importance of having sufficiently complete data in the years leading up to the period window, which we refer to as the pre-window. In the illustrated example, the pre-window spans 2012–2015 and the period window spans 2015–2017. When the pre-window is removed, as shown in Fig. 5, the variation in estimated marginal relative survival among the different imputation approaches is increased compared to that in Fig. 4. This is particularly notable for the estimates that also deviated in the presence of the pre-window, as labelled in Fig. 5. It is unsurprising that these estimates are the most sensitive to changes in the short-term stage distribution. All subjects diagnosed before the pre-window are required to have survived at least 3 years in order to have experienced follow-up in the period window. Hence, assuming localised stage at diagnosis here is moderately reasonable, and conversely assuming distant stage at diagnosis is less realistic. On the other hand, without the pre-window, imputation condition 3 assumes a subject diagnosed in 2014 with unknown stage at diagnosis who only survived for 1 year had localised cancer at the time of diagnosis. This is unlikely to be true in most cases.

Stage at diagnosis information was assumed to be missing for a further 20% of patients who were diagnosed in the pre-window and period window. For many cancer sites, more up-to-date recording of stage at diagnosis information has a higher proportion of completeness. Given the effectiveness of the methods being implemented here while using artificially higher proportions of missing data, it is indicative that the same approach will be equally as effective in cancer registry data where stage at diagnosis information is more complete. However, the missing data mechanism here is assumed to be missing completely at random, which is unlikely to be the case in registry data sets. In this real-world data, the missing data mechanism can be dependent on other patient-level variables, such as age, comorbidities and calendar year of diagnosis. The pattern of missingness alters over calendar time, and this can be influenced by a range of factors. Historically, stage at diagnosis information was not collected routinely and therefore completeness is likely to be poor, yet the underlying mechanism is more likely to be closer to missing at random. The improvement in routine collection of stage at diagnosis information as calendar time progresses suggests that any missing data still found is potentially due to a systematic process; however, this process may be unrelated to the healthcare system. The impact of calendar year on the variation in the missing data mechanism for stage at diagnosis can be lessened by applying a period analysis and pre-window period, as described throughout this paper. For patients diagnosed prior to the pre-window, the missing data mechanism was generated by conditioning on both stage at diagnosis and year of diagnosis. This could have been made more complex by also considering interaction effects between covariates.

We chose to use flexible parametric excess mortality models to estimate marginal relative survival due to their ability to capture complex baseline excess hazard functions, as well as covariate effects. The effect of stage varies greatly over time since diagnosis, hence we opted to fit models separately to each stage group, as opposed to fitting interaction effects between the effect of stage and time. Stratifying by stage also allows for the other covariate effects to vary by the three stage groups. We compared the estimates obtained by modelling the effect of stage at diagnosis by including it as a covariate effect and by stratifying across stage at diagnosis with the non-parametric estimates found in Fig. A1. We found a higher level of agreement using the stratification approach. In this paper, we provide marginal estimates; however, it is also possible to obtain covariate-specific estimates if desired.

Breast cancer tends to have a better prognosis and be diagnosed at an earlier stage than both colon and lung cancer. Additionally, the prognosis of localised and regional cases is far better than that of distant cases. The stage distribution is illustrated in Fig. C2. Compared to the colon and lung cohort as shown in Fig. 1 and Fig. B2, respectively, there are far more localised cases and far less distant cases. The stage-specific marginal relative survival estimates are given in Fig. C3. The agreement among the four imputation conditions is very good for the localised and regional estimates. The distant-stage marginal relative survival estimate differs when we apply imputation condition 4. Here, we assume all patients with missing cancer stage at diagnosis were diagnosed with distant breast cancer. This assumption is more extreme for breast cancer than colon or lung since patients are generally less likely to have distant cancer at the time of diagnosis.

There are other factors beyond those considered in this analysis that may impact cancer survival and need to be adjusted for in other settings, such as socio-economic status and geographical area. In extreme cases where a single region is more complete, then this approach inherently borrows information from that region for the long-term excess mortality rates. For example in England, the East of England reports lower proportions of missing stage information than other regions [22]. If there were further large regional differences in stage-specific survival, then accounting for region would be important. The proposed imputation model here is complex but exists to illustrate that the assumptions made regarding this imputation are not too impactful on the approach of borrowing strength from historical complete data.

## Conclusion

Long-term survival estimates are important in order to gain a greater understanding of the burden of cancer on society. Stage at diagnosis is a key prognostic factor but often poorly recorded historically. However, if the recent calendar period is sufficiently complete, robust stage-specific long-term survival estimates can be obtained using multiple imputation in the presence of a period analysis.

## Supplementary information

aj-checklist

Appendix

## Data Availability

All data used in this paper (individual case listings as well as US population mortality data) may be accessed and analysed via the SEER*Stat web program following the submission of a request for access to the data at https://seer.cancer.gov/seertrack/data/request/.

## References

[1] McPhail S, Johnson S, Greenberg D, Peake M, Rous B (2015). Stage at diagnosis and early mortality from cancer in England. Br J Cancer.

[2] Public Health England. Cancer survival in England for patients diagnosed between 2014 and 2018, and followed up until 2019. 2020. https://www.gov.uk/government/statistics/cancer-survival-in-england-for-patients-diagnosed-between-2014-and-2018-and-followed-up-until-2019. Accessed 9 Aug 2021.

[3] Pilleron S, Charvat H, Araghi M, Arnold M, Fidler-Benaoudia MM, Bardot A (2020). Age disparities in stage-specific colon cancer survival across seven countries: an ICBP SURVMARK-2 population-based study. Int J Cancer.

[4] Araghi M, Arnold M, Rutherford MJ, Guren MG, Cabasag CJ, Bardot A (2021). Colon and rectal cancer survival in seven high-income countries 2010–2014: variation by age and stage at diagnosis (the ICBP SURVMARK-2 project). Gut.

[5] Wills L, Pearson C. 10-year cancer survival by stage for patients diagnosed in the East of England, 2007 to 2017. CRUK-PHE partnership 2021 Feb. http://www.ncin.org.uk/about_ncin/10yearsurvival. Accessed 1 Oct 2021.

[6] Smith AJ, Lambert PC, Rutherford MJ (2021). Understanding the impact of sex and stage differences on melanoma cancer patient survival: a SEER-based study. Br J Cancer.

[7] Andersson TM, Dickman PW, Eloranta S, Lambe M, Lambert PC (2013). Estimating the loss in expectation of life due to cancer using flexible parametric survival models. Stat Med.

[8] Eisemann N, Waldmann A, Katalinic A (2011). Imputation of missing values of tumour stage in population-based cancer registration. BMC Med Res Methodol.

[9] Falcaro M, Nur U, Rachet B, Carpenter JR (2015). Estimating excess hazard ratios and net survival when covariate data are missing: strategies for multiple imputation. Epidemiology.

[10] Falcaro M, Carpenter JR (2017). Correcting bias due to missing stage data in the non-parametric estimation of stage-specific net survival for colorectal cancer using multiple imputation. Cancer Epidemiol.

[11] Luo Q, Egger S, Yu XQ, Smith DP, O’Connell DL (2017). Validity of using multiple imputation for “unknown” stage at diagnosis in population-based cancer registry data. PLoS ONE.

[12] Barclay ME, Lyratzopoulos G, Greenberg DC, Abel GA (2018). Missing data and chance variation in public reporting of cancer stage at diagnosis: cross-sectional analysis of population-based data in England. Cancer Epidemiol.

[13] Muller P, Woods L, Walters S (2020). Temporal and geographic changes in stage at diagnosis in England during 2008–2013: a population-based study of colorectal, lung and ovarian cancers. Cancer Epidemiol.

[14] Brenner H, Gefeller O (1996). An alternative approach to monitoring cancer patient survival. Cancer.

[15] Yu XQ, Baade PD, O’Connell DL (2012). Conditional survival of cancer patients: an Australian perspective. BMC Cancer.

[16] Janssen-Heijnen ML, van Steenbergen LN, Steyerberg E, Visser O, De Ruysscher DK, Groen HJ (2012). Long-term excess mortality for survivors of non-small cell lung cancer in the Netherlands. J Thorac Oncol.

[17] Surveillance, Epidemiology, and End Results (SEER) Program. SEER*Stat Database: incidence - SEER Research Data, 9 Registries, Nov 2020 Sub (1975-2018), National Cancer Institute, DCCPS, Surveillance Research Program, released April 2021, based on the November 2020 submission. www.seer.cancer.gov. Accessed 18 Jan 2022.

[18] Brenner H, Gefeller O, Hakulinen T (2004). Period analysis for ‘up-to-date’cancer survival data: theory, empirical evaluation, computational realisation and applications. Eur J Cancer.

[19] Ruhl J, Adamo M, Dickie L. SEER program coding and staging manual 2016: section V. Bethesda, MD: National Cancer Institute, 2016.

[20] Madley-Dowd P, Hughes R, Tilling K, Heron J (2019). The proportion of missing data should not be used to guide decisions on multiple imputation. J Clin Epidemiol.

[21] Araghi M, Fidler-Benaoudia M, Arnold M, Rutherford M, Bardot A, Ferlay J, et al. International differences in lung cancer survival by sex, histological type and stage at diagnosis: an ICBP SURVMARK-2 Study. Thorax. 2021;77:378–90. 10.1136/thoraxjnl-2020-216555 34282033

[22] CancerData. 2021. https://www.cancerdata.nhs.uk/stage_at_diagnosis. Accessed 17 Feb 2022.

[23] Cancer Registry of Norway. Cancer in Norway 2019 - cancer incidence, mortality, survival and prevalence in Norway. Oslo: Cancer Registry of Norway, 2020.

[24] Carpenter J, Kenward M. Multiple imputation and its application. Hoboken, NJ: John Wiley & Sons; 2012.

[25] White IR, Royston P (2009). Imputing missing covariate values for the Cox model. Stat Med.

[26] Keogh RH, Morris TP (2018). Multiple imputation in Cox regression when there are time‐varying effects of covariates. Stat Med.

[27] Lambert P. STPM2: Stata module to estimate flexible parametric survival models. 2010. https://ideas.repec.org/c/boc/bocode/s457128.html. Accessed 13 September 2021.

[28] Syriopoulou E, Mozumder SI, Rutherford MJ, Lambert PC (2019). Robustness of individual and marginal model-based estimates: a sensitivity analysis of flexible parametric models. Cancer Epidemiol.

[29] Lambert PC. standsurv. 2019. https://pclambert.net/software/standsurv/. Accessed 26 Aug 2021.

[30] Rubin DB. Multiple imputation for survey nonresponse. 1987. Accessed 19 March 2021.

[31] Lambert P. STPP: Stata module to compute Pohar-Perme non-parametric estimate of marginal relative (net) survival. 2020. https://ideas.repec.org/c/boc/bocode/s458743.html. Accessed 15 September 2021.

[32] Perme MP, Stare J, Estève J (2012). On estimation in relative survival. Biometrics.

[33] Corazziari I, Quinn M, Capocaccia R (2004). Standard cancer patient population for age standardising survival ratios. Eur J Cancer.

[34] StataCorp. Stata statistical software: release 17. College Station, TX: StataCorp LLC; 2021.

