# Supplementary material for: Obtaining long-term stage-specific relative survival estimates in the presence of incomplete historical stage information
Source: Br J Cancer. 2022 Jun 17;127(6):1061–8. doi: 10.1038/s41416-022-01866-8 (PMC9470741; doi:10.1038/s41416-022-01866-8)
Supplement: Supplementary file 2 — Appendix [file 41416_2022_1866_MOESM2_ESM.docx]

# Appendix

## A: Colon cancer

Table A1: Stage-specific marginal relative survival estimates at 1-year, 5-years and 10-years post-diagnosis for colon cancer patients who experienced follow-up in the period window (2015-2017). (1)-(4) refer to imputation conditions (1)-(4), with the pre-window from 2012-2015. † Full dataset refers to estimates obtained from the underlying dataset with no simulated missing data, and where pre-existing missing stage information is imputed.

|  | Localised | | | Regional | | | Distant | | |
| --- | --- | --- | --- | --- | --- | --- | --- | --- | --- |
|  | 1-year | 5-year | 10-year | 1-year | 5-year | 10-year | 1-year | 5-year | 10-year |
| Full Dataset^†^ | 0.937 | 0.875 | 0.810 | 0.898 | 0.721 | 0.631 | 0.508 | 0.156 | 0.092 |
| Multiple Imputation (1) | 0.933 | 0.864 | 0.806 | 0.892 | 0.705 | 0.609 | 0.495 | 0.146 | 0.082 |
| Complete Case (2) | 0.936 | 0.863 | 0.808 | 0.894 | 0.711 | 0.626 | 0.495 | 0.146 | 0.076 |
| All Localised (3) | 0.932 | 0.857 | 0.774 | 0.894 | 0.711 | 0.626 | 0.495 | 0.146 | 0.076 |
| All Distant (4) | 0.936 | 0.863 | 0.808 | 0.894 | 0.711 | 0.626 | 0.494 | 0.180 | 0.155 |

Figure A1: Estimated marginal relative survival of the colon cancer patients who experienced follow-up during the period window (2015-2017), with artificially increased missing stage information and pre-window (2012-2015). The solid lines denote estimates obtained using flexible parametric models and the dashed lines denote estimates obtained using the non-parametric Pohar-Perme approach. (1)-(4) refer to imputation conditions (1)-(4).

## B: Lung cancer

Table B1: Baseline characteristics for the whole cohort of lung cancer patients diagnosed from 2005-2017, prior to applying a period analysis. ^†^Stage proportions calculated from the observed distribution.

|  | **Sex** | | | | | |
| --- | --- | --- | --- | --- | --- | --- |
|  | **Female** | | **Male** | | **Total** | |
| **Total** | 98,146 | (48.38%) | 104,711 | (51.62%) | 202,857 | (100.00%) |
| **Age Group at Diagnosis** |  |  |  |  |  |  |
| **<45** | 1,554 | (1.58%) | 1,476 | (1.41%) | 3,030 | (1.49%) |
| **45-54** | 8,108 | (8.26%) | 7,876 | (7.52%) | 15,984 | (7.88%) |
| **55-64** | 20,512 | (20.90%) | 23,839 | (22.77%) | 44,351 | (21.86%) |
| **65-74** | 30,717 | (31.30%) | 34,271 | (32.73%) | 64,988 | (32.04%) |
| **>75** | 37,255 | (37.96%) | 37,249 | (35.57%) | 74,504 | (36.73%) |
| **Stage at Diagnosis^†^** | *(n=95,349)* |  | *(n=101,822)* |  | *(n=197,171)* |  |
| **Localised** | 20,624 | (21.63%) | 18,018 | (17.70%) | 38,642 | (19.60%) |
| **Regional** | 21,723 | (22.78%) | 23,451 | (23.03%) | 45,174 | (22.91%) |
| **Distant** | 53,002 | (55.59%) | 60,353 | (59.27%) | 113355 | (57.49%) |
|  |  |  |  |  |  |  |
| **Missing** | 2,797 | (2.85%) | 2,889 | (2.76%) | 5,686 | (2.80%) |
|  |  |  |  |  |  |  |
| **Mean (SD) Age at Diagnosis** |  |  |  |  |  |  |
| **Stage at Diagnosis** |  |  |  |  |  |  |
| **Localised** | 70.52 | (11.18) | 71.19 | (10.58) | 70.83 | (10.91) |
| **Regional** | 69.50 | (11.10) | 69.38 | (10.66) | 69.44 | (10.88) |
| **Distant** | 69.83 | (11.76) | 69.10 | (11.26) | 69.44 | (11.50) |

Figure B1: Estimated marginal relative survival (a) and excess hazard (b) for the full cohort of lung cancer patients diagnosed from 2005-2017.

Figure B2: Stage distribution among lung cancer patients observed over time for (a) the whole cohort diagnosed from 2005-2017, (b) the whole cohort with increased missing stage information, (c) the period cohort (period window 2015-2017) and (d) altered period cohort with increased missing stage information.

Figure B3: Estimated stage-specific marginal relative survival with pre-window (2012-2015) for lung cancer patients who experienced follow-up during the period window (2015-2017). (1)-(4) refer to imputation conditions (1)-(4).

## C: Breast cancer

Table C1: Baseline characteristics for the whole cohort of breast cancer patients diagnosed from 2005-2017, prior to applying a period analysis. ^†^Stage proportions calculated from the observed distribution.

| **Total** | 248,358 | (100.00%) |
| --- | --- | --- |
| **Age Group at Diagnosis** |  |  |
| **<45** | 28,597 | (11.51%) |
| **45-54** | 54,508 | (21.95%) |
| **55-64** | 64,181 | (25.84%) |
| **65-74** | 55,265 | (22.25%) |
| **>75** | 45,807 | (18.44%) |
| **Stage at Diagnosis^†^** | *(n=245,763)* |  |
| **Localised** | 160,132 | (65.16%) |
| **Regional** | 69,602 | (28.32%) |
| **Distant** | 16,029 | (6.52%) |
|  |  |  |
| **Missing** | 2,595 | (1.04%) |
|  |  |  |
| **Mean (SD) Age at Diagnosis** |  |  |
| **Stage at Diagnosis** |  |  |
| **Localised** | 62.17 | (13.34) |
| **Regional** | 58.69 | (14.15) |
| **Distant** | 61.64 | (14.79) |

Figure C1: Estimated marginal relative survival (a) and excess hazard (b) for the full cohort of breast cancer patients diagnosed from 2005-2017.

Figure C2: Stage distribution among breast cancer patients observed over time for (a) the whole cohort diagnosed from 2005-2017, (b) the whole cohort with increased missing stage information, (c) the period cohort (period window 2015-2017) and (d) altered period cohort with increased missing stage information.

Figure C3: Estimated stage-specific marginal relative survival with the pre-window (2012-2015) for breast cancer patients who experienced follow-up during the period window (2015-2017). (1)-(4) refer to imputation conditions (1)-(4).
